# Association between Ocular Parameters and Intraocular Pressure Elevation during Femtosecond Laser-Assisted Cataract Surgery in Open-Angle Glaucoma and Nonglaucoma Individuals

**DOI:** 10.3390/jpm12020257

**Published:** 2022-02-10

**Authors:** Ya-Hui Wang, Yi-Zhen He, Ming-Hsuan Chiang, Chia-Yi Lee, Chien-Liang Wu

**Affiliations:** 1 Department of Ophthalmology, Taipei Municipal Wanfang Hospital, Taipei 116081, Taiwan; judy5340@gmail.com (Y.-H.W.); 106185@w.tmu.edu.tw (Y.-Z.H.); 104129@w.tmu.edu.tw (M.-H.C.); 2 College of Medicine, Taipei Medical University, Taipei 110301, Taiwan; 3 Department of Ophthalmology, Beauty-Bright Eye Clinics, Taipei 106070, Taiwan; 4 Department of Ophthalmology, Nobel Eye Institute, Taipei 106074, Taiwan

**Keywords:** femtosecond laser-assisted cataract surgery, fluid-filled interface, axial length, intraocular pressure, open-angle glaucoma

## Abstract

In this study, we evaluate the association between biometrics and intraocular pressure (IOP) during femtosecond laser-assisted cataract surgery (FLACS) in normal patients and those with open-angle glaucoma (OAG). A retrospective cross-sectional study was conducted. A total of 103 patients who had received elective FLACS were enrolled, and those with OAG who received FLACS were further divided into a subgroup. The perioperative IOP of FLACS was measured before, during, and after the suction procedure. Demographic data and preoperative biometrics were collected from the medical records. The generalized linear model was applied to yield the adjusted odds ratio (aOR) and corresponding 95% confidence interval (CI) of each biometric for the IOP elevation in the whole group and the OAG subgroup. The mean preoperative IOP was 20.96 ± 4.79 mmHg, which rose to 55.37 ± 11.58 mmHg during suction, and decreased to 23.75 ± 6.42 mmHg after suction; the IOP both during and after suction was significantly higher than the presuction IOP (both *p* < 0.001). The mean IOP elevation was 34.41 ± 9.70 mmHg in the whole study population, and the difference in IOP elevation between OAG and nonglaucoma subgroups was not significant (*p* = 0.159). In the whole group, the presuction IOP, postdilated pupil size (PPS), and central corneal thickness (CCT) were positively corrected to higher IOP elevation (all *p* < 0.05), while axial length (AL) was negatively related to IOP elevation (aOR: 0.020, 95% CI: 0.008–0.699, *p* = 0.042). For the OAG subgroup, the longer AL was more significantly correlated to lower IOP elevation compared to those without glaucoma (aOR: 0.231, 95% CI: 0.106–0.502, *p* = 0.006). In conclusion, presuction IOP, PPS, and CCT are related to higher IOP during FLAC, while the AL is negatively correlated to the IOP elevation in FLACS, especially for patients with OAG. Reviewing these parameters before FLACS may enable physicians to find patients who are at risk of IOP elevation.

## 1. Introduction

Femtosecond laser-assisted cataract surgery (FLACS), first introduced in 2008, is widely applied for patients who need cataract surgery to improve visual acuity [1]. Corneal incision, capsulotomy, and lens fragmentation can be automatically managed by FLACS, which facilitates the performance of cataract surgery [1,2]. In addition, the use of FLACS can reduce corneal edema and endothelial cell loss compared to conventional cataract surgery in the early phase [3]. However, certain complications can occur in FLACS, including anterior capsular tear, posterior capsular rupture, suprachoroidal hemorrhage, and the elevation of intraocular pressure (IOP) [4,5,6]. 

For the mechanism of IOP elevation in FLACS, stabilizing the eye during FLACS with fixation devices is a necessary step, such as with laser in situ keratomileusis, and may lead to the transient elevation of IOP [7,8]. The corneal curved applanation interface changes the corneal curvature and leads to a huge amount of IOP surge, which can reach 30 mmHg on average [9]. Although the fluid-filled interface and curved interface with a soft contact lens produced a numerically lower IOP elevation than the corneal curved applanation interface did, the IOP spike still developed [10,11]. Since intraoperative reverse pupillary blocks may occur during FLACS [12], identifying patients with potential risk factors for developing elevated IOP other than instrument etiology cannot be overemphasized.

Regarding the correlation between elevated IOP and other ocular parameters in cataract surgery, the mean preoperative IOP and deep anterior chamber depth (ACD) were correlated to the postoperative IOP in conventional cataract surgery [13], and can be used as a predictive formula of IOP elevation [14]. Preoperative IOP was an important predictor for IOP elevation during FLACS in recent studies [15,16]. However, the above studies focused on the IOP status [13,14,15,16], and the influence of other ocular biometrics has seldom been investigated. Moreover, few reports about the same issue on patients with open-angle glaucoma (OAG) have been conducted compared to angle closure patients [13,14]. Since biometrics such as central corneal thickness (CCT) are different between glaucoma and normal subjects [17], the influence of biometrics on IOP elevation during FLACS between OAG and nonglaucoma patients may be different, and this needs validation.

The aim of our study is to evaluate the potential predictive factors, mainly ocular biometrics, for increasing intraoperative IOP in patients who have received FLACS using the fluid-filled interface. In addition, the potential different effect of ocular biometrics on IOP elevation during FLACS between OAG and nonglaucoma patients was analyzed.

## 2. Materials and Methods

### 2.1. Subject Selection

A retrospective cross-sectional study was conducted in the Taipei Municipal Wanfang Hospital. Patients scheduled for elective FLACS and intraocular lens implantation who were over 20 years old were enrolled in the study population. The exclusion criteria were: (1) angle-closure glaucoma or uncontrolled OAG with an IOP more than 21 mmHg despite medical treatment; (2) active inflammation in the eye; (3) a history of prominent corneal disease or corneal surgery, except superficial corneal injury; (4) a CCT less than 500 mm or greater than 600 mm; (5) active iris neovascularization; (6) the presence of intraocular silicone oil; or (7) ocular or systemic steroid use three months before the preoperative visit. After the selection process, a total of 103 patients who had undergone FLCS were included. In the study population, 12 patients were diagnosed with OAG and constituted the OAG subgroup in the statistical analysis.

### 2.2. Surgical Device and Technique

**Figure 1:**
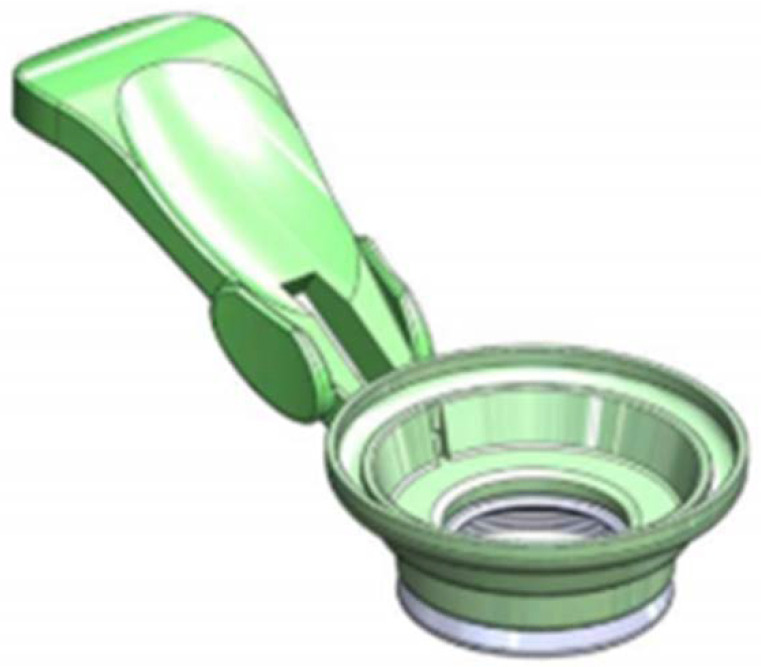
Schematic diagram of suction ring.

**Figure 2:**
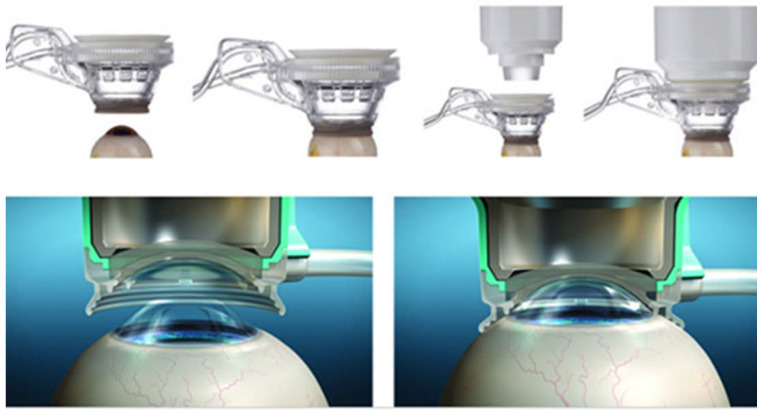
Schematic diagram of the placement of the suction ring on the corneal surface.

All FLACS procedures were performed in one eye of each patient by two well-trained surgeons (Chien-Liang Wu and Yi-Zhen He) with the same femtosecond laser system (LensAR Inc., Orlando, FL, USA). The patient interface of LensAR was composed of two parts: a suction ring that contacted only the sclera with an outer diameter of 18.0 mm and a disposable lens that rigidly mated the suction ring to the surgical system. In the first step, the suction ring (Figure 1) is centrally placed on the sclera of the eye (Figure 2). Briefly, after pupil dilation and topical corneal anesthesia, LensAR automatically detects the cornea, anterior chamber, and lens surfaces using Scheimpflug imaging and creates a 3-dimensional treatment plan. Then, the suction ring vacuum is enabled, which connects the suction ring to the patient’s eye; then, the suction ring volume is filled with a balanced salt solution. In the final step, the filled suction ring is adjusted and connected to the disposable lens. Excess fluid is free to discharge from any of three openings near the top of the suction-ring housing. The LensAR device created a 5.0 mm diameter curvilinear capsulotomy (4 mJ pulse energy), while the lens was segmented into quadrants and softened with grid spacing of 350 mm (10 mJ pulse energy). Upon the completion of the femtosecond procedure, the phacoemulsification procedure was subsequently performed by one device (INFINITI^®^ Vision System, Alcon, Fort Worth, TX, USA) in all patients. Directly after surgery, patients were asked whether they had vision problems during the procedure, and symptoms were recorded if they had occurred. Topical levofloxacin eyedrops and prednisolone emulsion were prescribed postoperatively four times per day for one week.

### 2.3. Main Outcome Measurement

Demographic data and medical history were obtained from the medical records. Preoperative biometrics, including ACD, CCT, and axial length (AL) were obtained by Optical Biometer AL-Scan (Nidek, Co., Ltd., Gamagori, Japan). Postdilated pupil size (PPS) was recorded after dilatation using the FLACS device. Regarding the IOP measurement, pre- and post-treatment IOPs were measured by another physician (Ya-Hui Wang) using a Tono-Pen^®^ XL (Reichert Inc., Buffalo, NY, USA), which was covered by individually wrapped Ocu-Film and tip covers before every application; then, the calibration was checked. Pre- and post-treatment IOP measurements were performed three times in each patient, and the average value was used, in which the time interval between the removal of the ring the post-treatment IOP measurement was within five seconds for all participants. For the IOP during the suction procedure, records were extracted from the FLACS device by the same surgeon (either Yi-Zhen He or Chien-Liang Wu). In addition, total suction time (ST) was collected from the same FLACS device. The above data were applied in subsequent analyses.

### 2.4. Statistical Analysis

SPSS version 20.0 (SPSS, Inc., Chicago, IL, USA) was utilized for statistical analysis in our study, except for power calculation. The statistical power of the OAG subgroup reached 0.73 under a 0.05 alpha value and medium effect size using G*power version 3.1.9.2 (Heinrich-Heine-Universität, Düsseldorf, Germany). Descriptive analysis was used to present the demography, systemic disease, existence of OAG, biometrics, ST, and IOP values, in which numerical indices are shown as the mean ± standard deviation (SD). Repeated one-way analysis of variance was used to compare the IOP value before, during, and after the FLACS procedure, and we constructed a box plot to demonstrate the distribution of IOP in each measurement. Then, the amount of IOP elevation was calculated via the following formula: IOP during FLACS minus the presuction IOP. In the next step, the generalized linear model was utilized to produce the adjusted odds ratio (aOR) and corresponding 95% confidence interval (CI) of the presuction IOP, PPS, and all the biometrics of the IOP elevation, which incorporated the effects of age, sex, eye laterality, systemic disease, the presence of OAG, and all ocular parameters. For subgroup analysis, the Mann–Whitney U test was used to compare the IOP value between the OAG and nonglaucoma subgroups. Furthermore, the generalized linear model was applied again to reveal the aOR and 95% CI of ocular parameters for IOP elevation in patients with OAG compared to nonglaucoma participants. For analysis with three or more values, we used Bonferroni correction/adjustment to refine the statistical results of multiple comparisons. A *p* value less than 0.05 was considered to indicate statistical significance, and a *p* value less than 0.001 was depicted as *p* < 0.001.

## 3. Results

The mean age of the study population was 66 ± 12.60 years old, and there were 52 male and 51 female patients in the study population. A total of 58 patients received FLACS for the right eye, and another 45 subjects received FLACS for the left eye. The details of systemic disease numbers and preoperative biometrics are shown in Table 1. 

**Figure 3:**
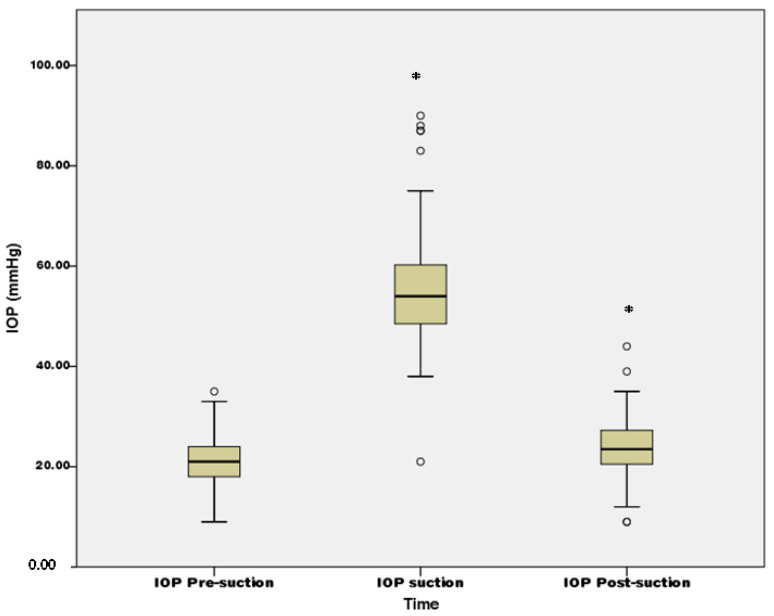
Box plot of intraocular pressure change during femtosecond laser-assisted cataract surgery, IOP: intraocular pressure, * denotes significant difference of intraocular pressure compared to presuction intraocular pressure.

The mean preoperative IOP was 20.96 ± 4.79 mmHg and rose to 55.37 ± 11.58 mmHg upon application of the suction ring and vacuum with significant difference (*p* < 0.001). After the removal of the suction ring, the mean IOP reduced to 23.75 ± 6.42 mmHg, which was significantly lower than the IOP during suction (*p* < 0.001), but still higher than that in the preoperative status (*p* < 0.001) (Figure 3). In addition, the amount of IOP elevation was 34.41 ± 9.70 mmHg in the whole study population. The OAG subgroup demonstrated a similar presuction IOP (23.91 ± 6.12 vs. 20.57 ± 4.49, *p* = 0.137), peak IOP (61.25 ± 14.78 vs. 54.60 ± 10.95, *p* = 0.168), and IOP elevation (37.33 ± 9.45 vs. 34.03 ± 9.71, *p* = 0.159) compared to nonglaucoma patients, while the postsuction IOP was significantly higher in the OAG subgroup (28.08 ± 6.66 vs. 23.18 ± 6.20, *p* = 0.037).

Regarding the correlation between ocular parameters and IOP elevation, higher presuction IOP (aOR: 2.399, 95%CI: 1.770–8.521, *p* = 0.038), larger PPS (aOR: 4.833, 95%CI: 2.418–9.660, *p* = 0.006), and thicker CCT (aOR: 5.551, 95%CI: 1.577–9.541, *p* = 0.012) were positively correlated to higher IOP elevation in the multivariable model. On the other hand, longer AL was correlated to lower IOP elevation (aOR: 0.020, 95%CI: 0.008–0.699, *p* = 0.042) after adjusting for all indices (Table 2). In subgroup analysis, longer AL was associated with lower IOP elevation in the OAG subgroup compared to the nonglaucoma population (aOR: 0.231, 95%CI: 0.106–0.502, *p* = 0.006). The effects of other parameters were similar between the two subgroups (all *p* > 0.05), and the effect of each parameter on IOP elevation in the two subgroups was similar to that in the whole group (Table 3).

## 4. Discussion

Briefly, our study demonstrates that an IOP elevation would occur after the performance of FLACS with a fluid-filled interface. The AL was negatively correlated to IOP elevation during FLACS, while presuction IOP, CCT, and PPS were positively related to IOP elevation. Moreover, the negative correlation between longer AL and IOP elevation was more prominent in OAG individuals compared to that in nonglaucoma patients.

Regarding the potential predicting factors for IOP elevation during FLACS, there was a relatively rare finding. In the study written by Liu et al., and Chang et al., both preoperative IOP and ACD were positively correlated to a higher postoperative IOP in patients with angle-closure glaucoma who had received traditional cataract surgery [13,14]. In another study written by Kerr et al., there was no significant association between IOP elevation and number of docking attempts, ST, treatment time, and CCT in patients receiving FLACS [8]. Two recent studies revealed significant association between preoperative IOP and IOP elevation during FLACS [15,16]. In the current study, the AL was negatively correlated to IOP elevation in patients who received FLACS. To our knowledge, this is a preliminary finding that suggests that a longer AL may prevent OP fluctuation in individuals receiving FLACS. A possible explanation is that the longer AL is also accompanied by a larger eyeball volume; thus, the force resulting from the suction procedure in FLACS might not prominently raise the IOP compared to those with smaller eyeball volume. Although the AL was associated with higher IOP in a previous epidemiological study [18], AL may be protective factor for external-pressure-induced IOP elevation in certain conditions. A previous study demonstrated that shorter AL was related to higher IOP elevation for those receiving intravitreal injection [19]. This may support our claim that a longer AL can reduce volume- or pressure-related IOP fluctuation.

Regarding other ocular parameters and the elevated IOP, presuction IOP, PPS, and CCT showed positively significant associations with higher IOP elevation in the current study. The effect of presuction IOP on the IOP elevation was established in previous studies [15,16], and our results further support the findings. A previous study showed that the IOP would rise and be sustained for about four hours after dilation [20]; thus, a large pupil may correlate to IOP spikes in patients receiving FLACS. On the other hand, the relationship between CCT and IOP elevation in the current study did not correspond to previous findings [8]. Since a thicker cornea is related to higher IOP measurement, which results from the resistance of ocular surface [21], it is possible that the thicker CCT may cause a higher peak IOP during the suction procedure. The mean CCT in our study population was 543.98 ± 35.09 µm, which is around the average of the general population [21]; thus, the IOP adjustment for CCT is unnecessary. The ACD did not reveal significant association with IOP elevation in FLACS in the current study, which contradicts previous results in which the ACD was regarded as a predictor of IOP elevation [13,14]. However, the patient population in the two previous studies comprised individuals with a close angle, and such individuals were not included in the current study [13,14]. The ACD may play a minor role in IOP elevation in patients with normal angle structure. The ST did not alter IOP fluctuation in our study, as no previous research reported this finding.

Research to evaluate the influence of ocular parameters on IOP elevation during FLACS in OAG individuals is rare. In the current study, the longer AL was associated with lower IOP elevation in OAG patients compared to nonglaucoma patients, and all the parameters showed a similar effect in the whole group, OAG subgroup, and nonglaucoma subgroup. In a previous study, OAG patients with an AL shorter than 26 mm were associated with larger 24-h IOP fluctuation [22]. No patients with OAG revealed an AL longer than 26 mm in our study population, but the results of that research may imply that IOP elevation may easily occur in patients with OAG and short AL. Since OAG tends to develop in patients with longer AL according to a population-based study [23], individuals with OAG and short AL might have certain factors that predispose them to IOP elevation and glaucoma progression. Further study is needed to confirm this hypothesis. Regarding the other parameters including presuction IOP, PPS, ACD, CCT and ST, the effect for IOP elevation is similar between OAG and nonglaucoma patients. Accordingly, the deep ACD may not be a contraindication of FLACS in those diagnosed with OAG. 

Patients with glaucoma would experience a higher elevation of IOP during FLACS compared to nonglaucomatous individuals, while the long-term effect of such IOP elevation is not fully elucidated [24]. In our study population, postsuction IOP was significantly higher in the OAG subgroup, while the amount of IOP elevation was not significantly different between the two subgroups, and no intraoperative pupillary block and malignant glaucoma occurred in the OAG subgroup. Moreover, the IOP in these OAG patients returned to a normal range—i.e., lower than 21 mmHg—within two weeks after the FLACS without additional medication or surgical intervention to slow the IOP. The above findings suggest the degree of IOP surge with fluid-filled interface is acceptable even in patients presenting with OAG. Furthermore, there were no OAG, angle closure glaucoma, normal tension glaucoma, or ocular hypertension events in our study population after the FLACS with a follow-up period up to 18 months. Consequently, the absence of newly developed glaucoma-related disorders in the current study may imply the safety of fluid-filled interface used FLACS [4]. 

For the interface device in FLACS, a flat applanating contact lens was first introduced for femtosecond laser flap creation [25]. This interface is still in use today despite very high IOP resulting from corneal compression during the applanation process, with the IOP surge potentially reaching above 90 mmHg [26]. To reduce these IOP peaks that may lead to acute glaucoma, a curved applanation interface that better fit onto the natural corneal curvature and reduced the maximum IOP to 65 mmHg during FLACS was utilized [21,25,27]. Recently, fluid-filled interfaces, such as immersion ultrasonic examination devices, have been introduced in the field of FLACS; these can minimize pressure on the cornea because there is no direct force or deformation of the corneal structure [28]. In our study, the mean IOP fluctuation was 34.41 mmHg, which was higher than the IOP alteration in previous studies using fluid-filled interfaces, which ranged from 10 to 30 mmHg during FLACS [25,26,29,30,31]. Nevertheless, the mean baseline IOP in the current study was 20.96 mmHg, which was also higher than the baseline IOP in previous studies [25,26,29,30,31] and may affect the IOP elevation during suction.

There are still limitations in our study. First, the absence of a control group made the comparison of different basic characters difficult. Second, we used tonopen only for the IOP measurement, while the application of a Pascal tonometer should be considered in subsequent research. Patient numbers between the OAG subgroups and the nonglaucoma subgroups were extremely uneven due to the retrospective design and could contribute to some statistical bias. The statistical power of the OAG subgroup was also relatively inadequate, yielding a power of 0.73. However, we also presented the effect of ocular parameters on IOP elevation in the general population; thus, we did not reduce the nonglaucoma participants, and recruiting some recent OAG cases who received FLACS may increase the heterogeneity of our study population concerning the time of surgery.

## 5. Conclusions

In conclusion, AL is negatively correlated to IOP elevation in patients receiving FLACS, especially for those with OAG. Furthermore, presuction IOP, PPS, and CCT are positively associated with IOP elevation in FLACS. Consequently, repeated examination of these parameters may be recommended for patients scheduled for FLACS, and the use of FLACS should be considered carefully in OAG patients with short AL. Further prospective study to quantify the influence of FLACS with a fluid-filled interface on different severities of glaucoma is mandatory.

## Figures and Tables

**Table 1:** Baseline characteristics of the study population.

Characteristics	Study Population (*N* = 103)
Age (years, mean ± SD)	66.72 ± 12.60
Sex (male/female)	52:51
Laterality (right/left)	58:45
Systemic disease (*N*) ^#^	
0	91
1	9
≥2	3
Glaucoma (*N*)	12
Ocular parameters (mean ± SD)	
Presuction IOP (mmHg)	20.96 ± 4.79
PPS (mm)	6.70 ± 0.74
ACD (mm)	2.89 ± 0.56
CCT (µm)	543.98 ± 35.09
AL (mm)	25.46 ± 2.70
ST (second)	154.68 ± 23.24

*N*: number, SD: standard deviation, IOP: intraocular pressure, PPS: postdilated pupil size, ACD: anterior chamber depth, CCT: central corneal thickness, AL: axial length, ST: suction time, ^#^ including hypertension, diabetes mellitus and chronic kidney disease.

**Table 2:** Correlation of each parameter to intraocular fluctuation during femtosecond laser-assisted cataract surgery.

Parameters	aOR	95% CI		*p* Value	
		Lower Limit	Upper Limit	Raw	Bonferroni Adjustment
Presuction IOP	2.399	1.770	8.521	0.023 *	0.038 *
PPS	4.833	2.418	9.660	<0.001 *	0.006 *
ACD	1.269	0.391	4.118	0.964	1.000
CCT	5.551	1.577	9.541	0.002 *	0.012 *
AL	0.020	0.008	0.699	0.037 *	0.042 *
ST	1.441	0.645	3.244	0.071	0.426

aOR: adjusted odds ratio, CI: confidence interval, IOP: intraocular pressure, PPS: postdilated pupil size, ACD: anterior chamber depth, CCT: central corneal thickness, AL: axial length, ST: suction time; * denotes significant correlation to intraocular fluctuation.

**Table 3:** Association of parameters with intraocular fluctuation during femtosecond laser-assisted cataract surgery in open-angle glaucoma compared to nonglaucoma individuals.

Parameters	aOR (95% CI)			*p* Value	
	OAG Subgroups (*N* = 12)	Nonglaucoma Subgroup (*N* = 91)	OAG to Nonglaucoma	Raw	Bonferroni Adjustment
Presuction IOP	2.687 (1.520–4.365) ^#^	2.225 (1.831–4.663) ^#^	1.084 (0.743–1.581)	0.677	0.993
PPS	6.477 (2.269–11.584) ^#^	3.912 (1.999–10.416) ^#^	1.860 (0.181–9.123)	0.602	0.834
ACD	1.752 (0.460–5.103)	1.093 (0.281–4.017)	2.421 (0.073–8.041)	0.621	0.918
CCT	5.823 (1.084–10.248) ^#^	5.485 (1.226–9.359) ^#^	1.029 (0.977–1.083)	0.278	0.552
AL	0.007 (0.001–0.176) ^#^	0.034 (0.009–0.752) ^#^	0.231 (0.106–0.502)	<0.001 *	0.006 *
ST	1.383 (0.534–3.160)	1.488 (0.669–3.833)	0.978 (0.906–1.056)	0.574	0.767

*N*: number, aOR: adjusted odds ratio, CI: confidence interval, IOP: intraocular pressure, PPS: postdilated pupil size, ACD: anterior chamber depth, CCT: central corneal thickness, AL: axial length, ST: suction time, ^#^ denotes significant correlation to intraocular fluctuation in that subgroup. * denotes significant correlation to intraocular fluctuation in open-angle glaucoma compared to nonglaucoma patients.

## Data Availability

The data are available upon reasonable request.

## References

[1] Abouzeid H., Ferrini W. (2014). Femtosecond-laser assisted cataract surgery: A review. Acta Ophthalmol..

[2] Wei Y., Xu L., Song H. (2017). Application of corvis st to evaluate the effect of femtosecond laser-assisted cataract surgery on corneal biomechanics. Exp. Ther. Med..

[3] Abell R.G., Kerr N.M., Howie A.R., Mustaffa Kamal M.A., Allen P.L., Vote B.J. (2014). Effect of femtosecond laser-assisted cataract surgery on the corneal endothelium. J. Cataract. Refract. Surg..

[4] Ebner M., Mariacher S., Januschowski K., Boden K., Seuthe A.M., Szurman P., Boden K.T. (2017). Comparison of intraocular pressure during the application of a liquid patient interface (femto ldv z8) for femtosecond laser-assisted cataract surgery using two different vacuum levels. Br. J. Ophthalmol..

[5] Ye Z., Li Z., He S. (2017). A meta-analysis comparing postoperative complications and outcomes of femtosecond laser-assisted cataract surgery versus conventional phacoemulsification for cataract. J. Ophthalmol..

[6] Roberts H.W., Day A.C., O’Brart D.P. (2020). Femtosecond laser-assisted cataract surgery: A review. Eur. J. Ophthalmol..

[7] Bissen-Miyajima H., Suzuki S., Ohashi Y., Minami K. (2005). Experimental observation of intraocular pressure changes during microkeratome suctioning in laser in situ keratomileusis. J. Cataract. Refract. Surg..

[8] Kerr N.M., Abell R.G., Vote B.J., Toh T. (2013). Intraocular pressure during femtosecond laser pretreatment of cataract. J. Cataract. Refract. Surg..

[9] Williams G.P., Ang H.P., George B.L., Liu Y.C., Peh G., Izquierdo L., Tan D.T., Mehta J.S. (2015). Comparison of intra-ocular pressure changes with liquid or flat applanation interfaces in a femtosecond laser platform. Sci. Rep..

[10] De Giacinto C., D’Aloisio R., Bova A., Candian T., Perrotta A.A., Tognetto D. (2019). Intraocular pressure changes during femtosecond laser-assisted cataract surgery: A comparison between two different patient interfaces. J. Ophthalmol..

[11] Kohnen T. (2013). Interface for femtosecond laser-assisted lens surgery. J. Cataract. Refract. Surg..

[12] Grewal D.S., Basti S. (2014). Intraoperative reverse pupillary block during femtosecond laser-assisted cataract surgery in a patient with phacomorphic angle closure. J. Cataract. Refract. Surg..

[13] Liu C.J., Cheng C.Y., Wu C.W., Lau L.I., Chou J.C., Hsu W.M. (2006). Factors predicting intraocular pressure control after phacoemulsification in angle-closure glaucoma. Arch. Ophthalmol. (Chic. Ill.: 1960).

[14] Chang Y.F., Ko Y.C., Lau L.I., Liu C.J. (2016). Verification of a formula developed to predict the postoperative intraocular pressure after cataract surgery in primary angle-closure glaucoma. J. Chin. Med. Assoc. JCMA.

[15] Mariacher S., Laubichler P., Mariacher M., Wendelstein J., Fischinger I., Bolz M. (2019). Impact of baseline iop, vacuum, and different docking mechanisms, and their interaction on iop rise in femtosecond laser-assisted refractive and cataract surgery. J. Cataract. Refract. Surg..

[16] Mariacher S., Laubichler P., Wendelstein J., Mariacher M., Bolz M. (2019). Preoperative intraocular pressure as a strong predictive factor for intraocular pressure rise during vacuum application in femtosecond laser-assisted cataract surgery. Acta Ophthalmol..

[17] Gaspar R., Pinto L.A., Sousa D.C. (2017). Corneal properties and glaucoma: A review of the literature and meta-analysis. Arq. Bras. Oftalmol..

[18] Foster P.J., Broadway D.C., Garway-Heath D.F., Yip J.L., Luben R., Hayat S., Dalzell N., Wareham N.J., Khaw K.T. (2011). Intraocular pressure and corneal biomechanics in an adult british population: The epic-norfolk eye study. Investig. Ophthalmol. Vis. Sci..

[19] Cacciamani A., Oddone F., Parravano M., Scarinci F., Di Nicola M., Lofoco G. (2013). Intravitreal injection of bevacizumab: Changes in intraocular pressure related to ocular axial length. Jpn. J. Ophthalmol..

[20] Kim J.M., Park K.H., Han S.Y., Kim K.S., Kim D.M., Kim T.W., Caprioli J. (2012). Changes in intraocular pressure after pharmacologic pupil dilation. BMC Ophthalmol..

[21] Kohlhaas M., Boehm A.G., Spoerl E., Pursten A., Grein H.J., Pillunat L.E. (2006). Effect of central corneal thickness, corneal curvature, and axial length on applanation tonometry. Arch. Ophthalmol. (Chicago, Ill.: 1960).

[22] Yang Y., Ng T.K., Wang L., Wu N., Xiao M., Sun X., Chen Y. (2020). Association of 24-hour intraocular pressure fluctuation with corneal hysteresis and axial length in untreated chinese primary open-angle glaucoma patients. Transl. Vis. Sci. Technol..

[23] Bikbov M.M., Gilmanshin T.R., Zainullin R.M., Kazakbaeva G.M., Arslangareeva I.I., Panda-Jonas S., Khikmatullin R.I., Aminev S.K., Nuriev I.F., Zaynetdinov A.F. (2020). Prevalence and associated factors of glaucoma in the russian ural eye and medical study. Sci. Rep..

[24] Darian-Smith E., Howie A.R., Abell R.G., Kerr N., Allen P.L., Vote B.J., Toh T. (2015). Intraocular pressure during femtosecond laser pretreatment: Comparison of glaucomatous eyes and nonglaucomatous eyes. J. Cataract. Refract. Surg..

[25] Talamo J.H., Gooding P., Angeley D., Culbertson W.W., Schuele G., Andersen D., Marcellino G., Essock-Burns E., Batlle J., Feliz R. (2013). Optical patient interface in femtosecond laser-assisted cataract surgery: Contact corneal applanation versus liquid immersion. J. Cataract. Refract. Surg..

[26] Sperl P., Strohmaier C., Kraker H., Motloch K., Lenzhofer M., Moussa S., Reitsamer H.A. (2017). Intraocular pressure course during the femtosecond laser-assisted cataract surgery in porcine cadaver eyes. Investig. Ophthalmol. Vis. Sci..

[27] Doughty M.J., Zaman M.L. (2000). Human corneal thickness and its impact on intraocular pressure measures: A review and meta-analysis approach. Surv. Ophthalmol..

[28] Furlanetto R.L., Facio A.C., Hatanaka M., Susanna Junior R. (2010). Correlation between central corneal thickness and intraocular pressure peak and fluctuation during the water drinking test in glaucoma patients. Clinics (Sao Paulo).

[29] Baig N.B., Cheng G.P., Lam J.K., Jhanji V., Chong K.K., Woo V.C., Tham C.C. (2014). Intraocular pressure profiles during femtosecond laser-assisted cataract surgery. J. Cataract. Refract. Surg..

[30] Ibarz M., Hernandez-Verdejo J.L., Bolivar G., Tana P., Rodriguez-Prats J.L., Teus M.A. (2016). Porcine model to evaluate real-time intraocular pressure during femtosecond laser cataract surgery. Curr. Eye Res..

[31] Schultz T., Conrad-Hengerer I., Hengerer F.H., Dick H.B. (2013). Intraocular pressure variation during femtosecond laser-assisted cataract surgery using a fluid-filled interface. J. Cataract. Refract. Surg..

